# Enteric Fever: Diagnostic Dilemma Encountered in Domperidone-Induced Neuroleptic Malignant Syndrome

**DOI:** 10.7759/cureus.15385

**Published:** 2021-06-02

**Authors:** Hawwa A Akhunzada, Hassan Rehman, Nabeel Tariq, Mohammad Ali Arif, Rauf Niazi

**Affiliations:** 1 Internal Medicine, Shaheed Zulfiqar Ali Bhutto Medical University/Pakistan Institute of Medical Sciences, Islamabad, PAK; 2 Internal Medicine and Endocrinology/Diabetology, Shaheed Zulfiqar Ali Bhutto Medical University/Pakistan Institute of Medical Sciences, Islamabad, PAK

**Keywords:** enteric fever (typhoid fever), neuroleptic malignant syndrome (nms), domperidone, drug-related side effects and adverse reactions, anti-emetics

## Abstract

Enteric fever is a multisystem illness caused by *Salmonella* Typhi and *Salmonella* Paratyphi, and it is associated with a spectrum of conditions ranging from minor cases of diarrhea and low-grade fever to a potentially fatal illness that can lead to gastrointestinal (GI) perforation, hemorrhage, central nervous system (CNS) involvement. Neuroleptic malignant syndrome (NMS) is predominantly described as an idiosyncratic reaction to neuroleptic medications. However, it has also been associated with a variety of drugs that reduce dopaminergic activity. In this report, we present the case of a young woman who had enteric fever and was prescribed IV ceftriaxone and domperidone. She subsequently developed NMS secondary to domperidone. We highlight the challenges faced when dealing with two potentially lethal medical conditions presenting concurrently and their overlapping symptoms. The patient was treated for both of the conditions and recovered completely.

## Introduction

Enteric fever constitutes a huge disease burden with an incidence rate of 976 per 100,000 people/year in the South Asian subcontinent, aggravating an already stretched healthcare scenario [1]. *Salmonella *Typhi is a major causative bacteria [2], causing approximately four times more cases than *Salmonella *Paratyphi A [3]. This illness can present as mild cases of fever and gastrointestinal (GI) disturbance but can also lead to serious consequences, and it has a case fatality rate of 2.4% [‎4]. Fever is identified as the major symptom of this condition [2-5]. Blood cultures are the gold standard method for the diagnosis of enteric fever [6].

Neuroleptic malignant syndrome (NMS) is a rare complication traditionally linked to antipsychotic medications and is clinically characterized by fever, generalized muscular rigidity, dysautonomia, and altered level of consciousness [7]. It has also been described secondary to other drugs that affect the dopaminergic pathway, including mood stabilizers, anti-depressants, as well as dopamine withdrawal following dopamine agonist treatment [7]. Domperidone has also been implicated as a causative agent [8-9]. Early recognition of NMS is critical since the condition has a high mortality rate [10].

We present a case of a young woman diagnosed with enteric fever based on blood cultures, who was initially started on empiric treatment including third-generation cephalosporin along with the antiemetic domperidone, which led to NMS that was diagnosed on the basis of clinical findings, raised creatine phosphokinase (CPK) and lactate dehydrogenase (LDH) levels, normal cerebrospinal fluid (CSF) analysis, normal brain imaging, and diffuse encephalopathy on electroencephalogram (EEG).

Informed consent was obtained from the patient for using her details for reporting purposes.

## Case presentation

A 25-year-old Pakistani female with no comorbid conditions presented to our Emergency Department in September 2020 with high-grade fever for one week, which had developed in a step-ladder fashion and was associated with nausea. She did not have ear discharge, cough, diarrhea, burning micturition, PV discharge, or meningism. On examination, she was febrile with a temperature of 101 °F, had a pulse rate of 94/minute, and a normal respiratory rate and blood pressure. Systemic examination was unremarkable. Initial investigations showed a total leucocyte count of 4,640 cells/µL with monocytosis, platelets of 140,000/µL, and hemoglobin of 10.5 g/dl (normocytic normochromic). Liver and renal function tests, as well as electrolytes, were within normal ranges (Table 1).

**Table 1:** Blood investigations

Variables	29/9/2020	1/10/2020	3/10/2020	4/10/2020	5/10/2020	6/10/2020	8/10/2020	10/10/2020	12/10/2020	Normal range
Complete blood count										
White blood cell count, /µL	4,640	2,970	3,550	3,940	3,940	2,780	3,850	4,000	3,820	4,000-1,100
Hemoglobin, g/dl	10.6	9.5	8.8	7.8	5.2	6.7	8	8.9	10.0	12-16
Platelets, /µL	140,000	67,000	83,000	80,000	64,000	57,000	101,000	131,000	199,000	150,000-400,000
Serum profile										
Lactate dehydrogenase, U/L			1,752	1,298	1,098	781	680	530	350	140-280
Creatine kinase, U/L			931	832	521	352	135	60	40	39-308
Urea, mg/dl			20	19	12	15	12	8	15	6-20
Creatinine, mg/dl	0.30		.29	.20	.22	.20	.25	.20	.22	0.5-1.1
Alanine transaminase, IU/L	61		170	115	101	90	56	55	53	29-33
Alkaline phosphatase, IU/L	92		72	114	161	185	141	151	136	44-147

Her blood cultures were drawn, and she was started empirically on third-generation cephalosporin based on the suspicion of enteric fever. She was also prescribed domperidone 10 mg thrice a day for nausea.

After four days of in-hospital stay, she developed decreased level of consciousness, generalized rigidity with dysarthria and dysphagia. She spiked a fever of 104 °F, had tachycardia at 120 beats/minute, tachypnea at 34 breaths/minute, blood pressure of 130/90, and dyspnea with SpO_2_ of 92% on room air. She was sweating profusely, and her chest expansion symmetrically decreased. There was a generalized lead pipe rigidity and bilateral hand tremors. The patient was drowsy but arousable.

NMS secondary to domperidone was diagnosed. Supporting evidence for NMS included elevated CPK at 931 U/L, LDH of 1,752 U/L (Table 1), a normal CSF fluid analysis (Table 2), and MRI brain findings [which ruled out meningoencephalitis as a central nervous system (CNS) complication of enteric fever]. CT brain was unremarkable as well. EEG showed background activity of diffuse low voltage rhythm suggestive of diffuse encephalopathy of metabolic degenerative or vascular origin. Her diagnosis was delayed due to the fact that spiking fever and decreased conscious level were initially attributed to her typhoid fever, and hence the management of NMS was also delayed. Once she developed rigidity and tremors, she was worked up further, upon which the diagnosis of NMS was made.

**Table 2:** Cerebrospinal fluid analysis CSF: cerebrospinal fluid

CSF routine examination	Results	Reference
Color	Colorless	Colorless
Turbidity	Clear	Clear
Red blood cells	Nil/mm^3^	Nil/mm^3^
White blood cells	4/mm^3^	Less than 5/mm^3^
Xanthochromia	Absent	Absent
Gram stain	No bacterial organism seen	None
Acid-fast bacilli stain	No acid-fast bacilli seen	None

The patient’s blood culture report received on the sixth day of admission showed extensively drug-resistant (XDR) *Salmonella* Typhi resistant to ceftriaxone but sensitive to azithromycin and meropenem. Her antibiotics were upgraded to azithromycin 500 mg IV once a day after a loading dose of 1 g and meropenem 1 g IV thrice a day.

For NMS, she was started on IV hydration, amantadine 100 mg twice a day, bromocriptine 2.5 mg twice a day, diazepam 5 mg twice a day, and benzhexol 1 mg once a day initially. She showed gradual response with improvement in rigidity, and three days later, her benzhexol and diazepam were discontinued. She was now kept on amantadine 100 mg thrice a day and bromocriptine 2.5 mg thrice a day.

She developed pancytopenia with a corrected reticulocyte count of 24,000 and normal RBC morphology on peripheral film. It was observed to be a complication of enteric fever, which responded to the antibiotics. The patient was discharged after 15 days of hospitalization with markedly improved lab parameters; she was afebrile and her rigidity had resolved.

## Discussion

Enteric fever has a nonspecific presentation that makes it challenging from a diagnostic perspective [11]. The initiation of antibiotics prior to adequate diagnostic investigations reduces the accuracy of the tests [2]. Blood cultures should be obtained prior to starting antibiotics and once blood cultures are reported, they should be dealt with accordingly. Third-generation cephalosporins such as ceftriaxone are recommended for commencing the treatment. However, in Pakistan, the majority of *Salmonella* Typhi isolates have developed antimicrobial resistance [12].

Disruption of the dopaminergic pathway in basal ganglia and hypothalamus caused either by dopamine receptor antagonism, decreased levels of the neurotransmitter, or by neuronal degeneration of dopaminergic fibers has been associated with the pathogenesis of NMS [7]. Domperidone is a dopamine receptor antagonist.

NMS in a patient already suffering from enteric fever may be confused as CNS complications of enteric fever, which can lead to a delay in reaching a correct diagnosis. Moreover, both conditions manifest fever. Physical examination and blood investigations like CPK and LDH [‎7] should be promptly performed to rule out extrapyramidal side effects of the offending agent. NMS can be treated with bromocriptine, dantrolene, amantadine, and there is some role for anticholinergics as well [7-14]. In cases of enteric fever, CNS complications must be ruled out in a timely manner before labeling the patient as having another illness due to drug side-effect profile. CNS complications can be easily ruled out by performing CSF analysis, conducting neuroimaging, and performing an EEG [15].

## Conclusions

Domperidone is generally considered a safe drug. However, its unusual side effect of NMS should not be missed. Patients with enteric fever manifest high-grade fever, and hence fever due to NMS can be easily missed out and a consequent delay in diagnosis can occur. Development of NMS should be suspected if there is an onset of autonomic dysfunction, rigidity, and delirium in a patient on offending drugs. Domperidone should be avoided as a treatment for gastroparesis or nausea in patients with enteric fever since they already might be dehydrated due to continuous or step-ladder fever and predisposed to NMS with its dopamine receptor antagonism.

## References

[1] Divyashree S, Nabarro LE, Veeraraghavan B, Rupali P (2016). Enteric fever in India: current scenario and future directions. Trop Med Int Health.

[2] Mukhopadhyay B, Sur D, Gupta SS, Ganguly NK (2019). Typhoid fever: control & challenges in India. Indian J Med Res.

[3] GBD 2017 Typhoid and Paratyphoid Collaborators (2019). The global burden of typhoid and paratyphoid fevers: a systematic analysis for the Global Burden of Disease Study 2017. Lancet Infect Dis.

[4] Pieters Z, Saad NJ, Antillón M, Pitzer VE, Bilcke J (2018). Case fatality rate of enteric fever in endemic countries: a systematic review and meta-analysis. Clin Infect Dis.

[5] Kumar P, Kumar R (2017). Enteric fever. Indian J Pediatr.

[6] Thriemer K, Ley B, Ame SS (2012). Clinical and epidemiological features of typhoid fever in Pemba, Zanzibar: assessment of the performance of the WHO case definitions. PLoS One.

[7] Tse L, Barr AM, Scarapicchia V, Vila-Rodriguez F (2015). Neuroleptic malignant syndrome: a review from a clinically oriented perspective. Curr Neuropharmacol.

[8] Spirt MJ, Chan W, Thieberg M, Sachar DB (1992). Neuroleptic malignant syndrome induced by domperidone. Dig Dis Sci.

[9] Kaur U, Chakrabarti SS, Gambhir IS (2015). Domperidone induced neuroleptic malignant syndrome: an uncommon toxicity of a common drug. Asian J of Pharmacol Toxicol.

[10] Samie MR (1987). Neuroleptic malignant-like syndrome induced by metoclopramide. Mov Disord.

[11] Gibani MM, Britto C, Pollard AJ (2018). Typhoid and paratyphoid fever: a call to action. Curr Opin Infect Dis.

[12] Qamar FN, Yousafzai MT, Dehraj IF (2020). Antimicrobial resistance in typhoidal salmonella: Surveillance for Enteric Fever in Asia Project, 2016-2019. Clin Infect Dis.

[13] Gurrera RJ, Caroff SN, Cohen A (2011). An international consensus study of neuroleptic malignant syndrome diagnostic criteria using the Delphi method. J Clin Psychiatry.

[14] Bakri YN, Khan R, Subhi J, Kawi Z (1992). Neuroleptic malignant syndrome associated with metoclopramide antiemetic therapy. Gynecol Oncol.

[15] Shaikh AIA, Prabhakar AT Typhoid fever and its nervous system involvement. Innate Immunity in Health and Disease.

